# AI-Based Quantification of Botulinum Neurotoxin-Induced Facial Changes: Wrinkle Reduction, Region-Specific Effects, and Functional Correlates of Facial Muscle Activity

**DOI:** 10.3390/toxins18040188

**Published:** 2026-04-15

**Authors:** Ibrahim Güler, Armin Kraus, Gerrit Grieb, Henrik Stelling

**Affiliations:** 1 Department of Plastic, Aesthetic and Hand Surgery, Otto-von-Guericke University, 39120 Magdeburg, Germany; armin.kraus@med.ovgu.de; 2 Department of Health Management, Friedrich-Alexander-Universität Erlangen-Nürnberg, Lange Gasse 20, 90403 Nürnberg, Germany; henrikstelling@googlemail.com; 3 Department of Plastic Surgery and Hand Surgery, Gemeinschaftskrankenhaus Havelhöhe, Kladower Damm 221, 14089 Berlin, Germany; gerritgrieb@gmx.de; 4 Department of Plastic Surgery and Hand Surgery, RWTH Aachen University, Pauwelsstrasse 30, 52074 Aachen, Germany; 5 Practices for Nuclear Medicine, Rubensstraße 125, 12157 Berlin, Germany

**Keywords:** botulinum neurotoxin, facial muscle activity, wrinkle assessment, neuromuscular modulation, aesthetic medicine, facial aging, treatment outcome assessment, plastic surgery, artificial intelligence, multimodal large language model

## Abstract

Botulinum neurotoxin (BoNT) treatment outcomes are commonly assessed through visual evaluation of facial wrinkle patterns, a process that remains inherently subjective despite structured grading systems. This study evaluated whether contemporary multimodal artificial intelligence (AI) systems can identify facial changes associated with BoNT treatment, using region-specific wrinkle patterns as surrogate markers of underlying muscle activity. A dataset of 46 facial images (23 pre-treatment, 23 post-treatment) was analyzed using four multimodal models, each assessed across five independent runs. Models were tasked with classifying treatment state from single images, detecting wrinkle presence in the forehead, glabella, and periorbital regions, and generating exploratory severity scores and age estimates. Two models achieved 100% accuracy in distinguishing pre- from post-treatment images in this dataset, while region-specific wrinkle detection was variable and frequently did not exceed majority-class baselines. Inter-run reliability varied substantially across models. Exploratory wrinkle severity scores showed directional differences between treatment states, whereas apparent age estimates demonstrated minimal systematic variation. These findings suggest that global facial changes associated with BoNT treatment appear to be detectable in model outputs, but region-specific assessment remains limited, underscoring the need for cautious interpretation and further validation.

## 1. Introduction

Botulinum neurotoxin (BoNT) is one of the most widely used agents in aesthetic medicine. By selectively inhibiting acetylcholine release at the neuromuscular junction, BoNT induces temporary chemodenervation of treated muscles, thereby reducing the appearance of dynamic wrinkles caused by repetitive facial muscle contraction [1,2,3].

The upper face represents the primary domain of aesthetic BoNT application, with established treatment indications for forehead lines, glabellar frown lines, and periorbital lines (lateral canthal rhytids). These three regions correspond directly to the frontalis, corrugator supercilii/procerus, and orbicularis oculi muscles, whose hyperactivity is the principal driver of age-related dynamic wrinkles in the upper face. In contrast, aesthetic concerns in the mid- and lower face are primarily addressed through soft tissue fillers, while BoNT plays a more secondary role [1,4,5,6].

Clinicians routinely assess static and dynamic wrinkle patterns across the forehead, glabella, and periorbital regions to guide treatment indication, dosing, and outcome evaluation. Although structured severity grading systems have improved objectivity and reproducibility, visual wrinkle assessment remains inherently subjective and dependent on examiner experience [1,5,7,8]. Recent conceptual frameworks, such as the Index of Muscular Equilibrium (IME), have further highlighted the importance of quantitative wrinkle and muscle activity assessment for individualized BoNT treatment planning [9].

Despite its clinical importance, BoNT outcome assessment remains largely subjective, with well-documented inter-rater variability even among experienced clinicians. This limits comparability across studies and underscores the need for observer-independent assessment tools, particularly in settings requiring consistent evaluation of subtle changes in wrinkle patterns and muscle activity [5,7,8].

To address these limitations, recent advances in artificial intelligence (AI), particularly in deep learning (DL) and transformer-based foundation models, following the seminal “Attention Is All You Need” publication (Vaswani et al., 2017), have enabled the development of multimodal large language models (MLLMs), a class of generative artificial intelligence (GenAI) systems capable of processing both text and image inputs [10,11,12,13]. As multimodal extensions of large language models (LLMs), MLLMs can integrate and interpret visual information and generate structured outputs, making them promising candidates for observer-independent assessment tasks. Recent evaluations have demonstrated variable MLLM performance on radiographic interpretation tasks, including fracture detection, dermatological assessment, and facial aging evaluation [12,13,14,15,16]. A recent study demonstrated that a contemporary MLLM achieved near-perfect agreement with board-certified plastic surgeons for facelift candidacy assessment based on facial photographs alone, suggesting that these models possess meaningful capacity for facial analysis [16].

However, a critical gap persists in the evaluation of BoNT treatment outcomes. While MLLMs have been increasingly evaluated across various medical image interpretation tasks, including broad facial aging assessment [11,12,16], their ability to detect visual patterns specifically consistent with BoNT-induced facial changes has rarely been examined. The effects of BoNT on upper-face wrinkle patterns are well characterized clinically: reduction or elimination of dynamic wrinkles in the forehead, glabella, and periorbital regions, accompanied by changes in apparent facial expression, perceived age, and overall muscular equilibrium [1,2,3,4,5,6,7,8,9]. These visually observable changes can be interpreted as a functional readout of BoNT–induced neuromuscular modulation, suggesting a non-invasive surrogate of altered facial muscle activity [1,17,18]. Whether contemporary multimodal AI systems can recognize these treatment-associated visual changes from single-image inference, without access to paired before/after comparisons or clinical history, remains largely unexplored.

The present study addresses this gap by evaluating whether four contemporary multimodal AI systems can classify facial images as consistent with pre- or post-BoNT treatment status, detect region-specific wrinkle presence in the forehead, glabella, and periorbital areas, and generate wrinkle severity ratings and apparent age estimates. Each image was evaluated independently across five runs to assess both classification accuracy and inter-run reliability [19,20,21].

## 2. Results

### 2.1. Dataset Characteristics

The dataset comprised 46 facial images with a balanced distribution of treatment states (23 BEFORE, 23 AFTER). Ground truth wrinkle annotations showed moderate to substantial class imbalance favoring the absent class across all three regions: forehead (30 absent, 16 present; majority-class proportion: 65.2%), glabella (34 absent, 12 present; 73.9%), and periorbital (36 absent, 10 present; 78.3%). Each image was evaluated by four MLLMs across five independent runs, yielding 920 model evaluations per task (46 images × 4 models × 5 independent runs; Table 1).

### 2.2. Treatment State Classification

**Figure 1:**
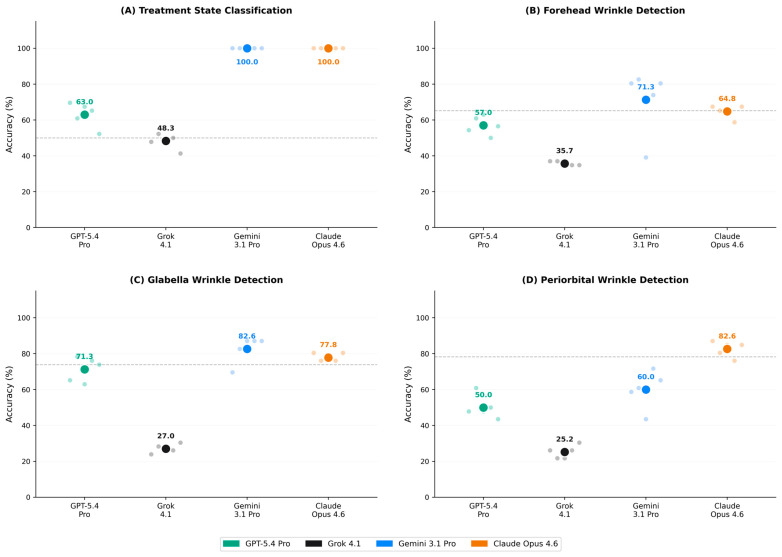
Multi-panel overview of classification accuracy across primary tasks. Each small translucent dot represents accuracy from one of five independent inference runs per model; large colored dots indicate mean accuracy across runs. (**A**) Treatment state classification; (**B**) forehead wrinkle detection; (**C**) glabella wrinkle detection; and (**D**) periorbital wrinkle detection. Horizontal dashed lines indicate the majority-class accuracy, i.e., the expected accuracy of a naive classifier predicting the most frequent class ((**A**): 50.0%; (**B**): 65.2%; (**C**): 73.9%; and (**D**): 78.3%). Model colors: GPT-5.4 Pro (green, OpenAI), Grok 4.1 (black, xAI), Gemini 3.1 Pro (blue, Google DeepMind), and Claude Opus 4.6 (orange, Anthropic).

Performance in treatment state classification (BEFORE vs. AFTER) varied markedly across models (Figure 1, Table 2). Gemini 3.1 Pro and Claude Opus 4.6 both achieved perfect accuracy (100.0%) with perfect inter-run agreement (κ = 1.000) across all five runs. GPT-5.4 Pro demonstrated intermediate performance (63.0 ± 6.9%; range: 52.2–69.6%) with fair inter-run reliability (κ = 0.389). Grok 4.1 performed below the majority-class proportion of 50.0% (48.3 ± 4.2%; range: 41.3–52.2%) with negative κ (−0.027), indicating agreement below chance level.

The sensitivity-specificity profiles revealed distinct classification biases. Grok 4.1 exhibited near-zero sensitivity for detecting AFTER images (0.9 ± 1.9%) but high specificity for BEFORE images (95.7 ± 7.5%), indicating a systematic bias toward classifying images as pre-treatment. GPT-5.4 Pro showed a similar asymmetry (sensitivity: 38.3 ± 9.9%; specificity: 87.8 ± 9.4%), suggesting a conservative classification tendency favoring the BEFORE label.

### 2.3. Region-Specific Wrinkle Detection

Wrinkle detection accuracy varied substantially across regions and models (Table 2). For forehead wrinkle detection (majority-class proportion: 65.2%), Gemini 3.1 Pro achieved the highest mean accuracy (71.3 ± 18.3%; range: 39.1–82.6%), though with notable inter-run variability. Claude Opus 4.6 demonstrated more stable performance (64.8 ± 3.6%) with the highest inter-run reliability in this task (κ = 0.808, almost perfect). GPT-5.4 Pro performed near the majority-class proportion (57.0 ± 5.2%), while Grok 4.1 fell substantially below it (35.7 ± 1.2%).

For glabella wrinkle detection (majority-class proportion: 73.9%), Gemini 3.1 Pro achieved the highest accuracy (82.6 ± 7.5%), followed by Claude Opus 4.6 (77.8 ± 2.4%). Both models exceeded the majority-class proportion, with substantial inter-run reliability (κ = 0.636 and 0.732, respectively). GPT-5.4 Pro performed near the majority-class proportion (71.3 ± 6.8%), while Grok 4.1 fell well below it (27.0 ± 2.5%).

For periorbital wrinkle detection (majority-class proportion: 78.3%), Claude Opus 4.6 achieved the highest accuracy (82.6 ± 4.3%) and was the only model to exceed the majority-class proportion, with moderate inter-run reliability (κ = 0.601). Gemini 3.1 Pro showed intermediate performance (60.0 ± 10.5%) below the majority-class proportion, and GPT-5.4 Pro performed at chance level (50.0 ± 6.5%). Grok 4.1 consistently fell below all majority-class proportions (25.2 ± 3.6%).

**Figure 2:**
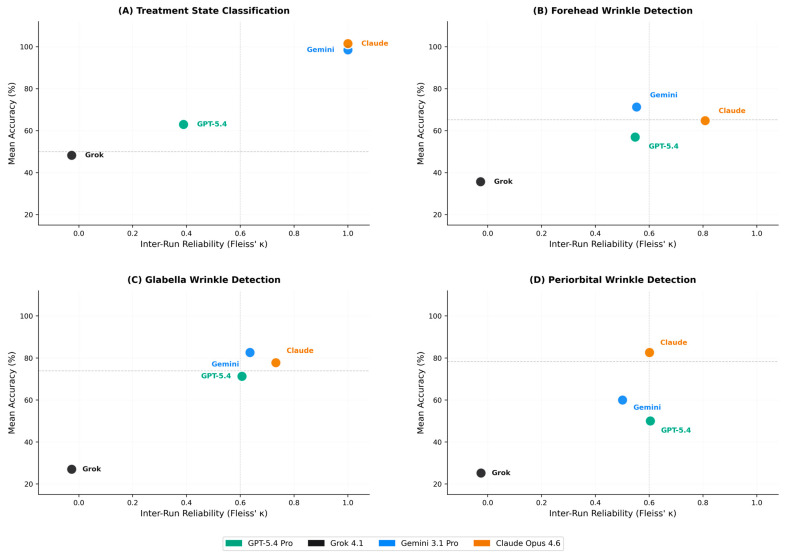
Task-specific accuracy–consistency profiles. Models are plotted according to mean classification accuracy (y-axis) and inter-run reliability measured by Fleiss’ κ (x-axis). Horizontal dashed lines indicate majority-class proportions (task-specific). Vertical dotted lines indicate κ = 0.60 as an indicative threshold for substantial agreement. (**A**) Treatment state classification; (**B**) forehead wrinkle detection; (**C**) glabella wrinkle detection; and (**D**) periorbital wrinkle detection. For treatment state classification (Panel A), Gemini 3.1 Pro and Claude Opus 4.6 both achieved κ = 1.000 and 100.0% accuracy; points are offset vertically for visibility. Model colors: GPT-5.4 Pro (green, OpenAI), Grok 4.1 (black, xAI), Gemini 3.1 Pro (blue, Google DeepMind), and Claude Opus 4.6 (orange, Anthropic). κ = Fleiss’ kappa [23].

Sensitivity–specificity analysis revealed that Grok 4.1 exhibited a systematic overdetection bias across all regions, with consistently high sensitivity (90.0–97.5%) but near-zero specificity (2.4–7.2%), indicating that this model classified nearly all images as wrinkle-present regardless of ground truth. GPT-5.4 Pro showed a similar, though less extreme, pattern. In contrast, Claude Opus 4.6 demonstrated the most balanced sensitivity–specificity profiles across regions (Figure 1 and Figure 2).

### 2.4. Summary Performance

Aggregated mean accuracy across the four binary classification tasks (each task equally weighted) ranged from 34.0% (Grok 4.1) to 81.3% (Claude Opus 4.6). Gemini 3.1 Pro achieved 78.5%, GPT-5.4 Pro 60.3%, and Grok 4.1 34.0% (Table 3). The ranking shifted depending on task: Gemini 3.1 Pro and Claude Opus 4.6 dominated treatment state classification, whereas Claude Opus 4.6 showed the most consistent performance across wrinkle detection tasks.

### 2.5. Accuracy–Consistency Dissociation

Fleiss’ κ analysis revealed marked differences in inter-run reliability across models (Table 2). Claude Opus 4.6 achieved the highest and most consistent reliability across all tasks (κ range: 0.601–1.000), with almost perfect agreement for treatment state and forehead wrinkle detection, and substantial to moderate agreement for glabella and periorbital tasks. Gemini 3.1 Pro showed perfect reliability for treatment state (κ = 1.000) but only moderate agreement for wrinkle detection tasks (κ = 0.501–0.636). GPT-5.4 Pro demonstrated fair to moderate reliability (κ = 0.389–0.606).

Grok 4.1 showed consistently negative or near-zero κ values across all tasks (κ range: −0.027 to −0.025), reflecting highly variable responses across runs. This pattern reflects that Grok 4.1 produced highly inconsistent responses across runs, with each inference yielding divergent outputs independent of prior assessments of the same image.

### 2.6. Exploratory Wrinkle Severity Scores

Model-predicted wrinkle severity scores (ordinal, 0–4) were stratified by true treatment state (Table 4). Three of four models assigned higher severity scores to BEFORE images compared with AFTER images across all three regions, consistent with the expected pattern of reduced wrinkle severity following BoNT treatment. The largest score differences were observed for Gemini 3.1 Pro (forehead Δ = +1.75; glabella Δ = +1.10; periorbital Δ = +1.07) and Claude Opus 4.6 (forehead Δ = +1.39; glabella Δ = +0.92). GPT-5.4 Pro showed moderate differences across regions (Δ = +0.99 to +1.32).

Grok 4.1 showed minimal score differentiation between treatment states across all regions (forehead Δ = +0.07; glabella Δ = +0.05; periorbital Δ = +0.27), consistent with its poor classification performance on binary tasks. Claude Opus 4.6 showed a notably small periorbital score difference (Δ = +0.11) despite achieving the highest binary detection accuracy for this region, suggesting that the model distinguished wrinkle presence without assigning proportionally differentiated severity ratings.

Inter-run reliability for ordinal scores followed the same model hierarchy as binary tasks. Claude Opus 4.6 achieved the highest κ values (0.521–0.781), followed by Gemini 3.1 Pro (0.414–0.484) and GPT-5.4 Pro (0.383–0.402). Grok 4.1 showed near-zero reliability (κ = −0.051 to 0.007).

### 2.7. Exploratory Apparent Age Estimates

Apparent age estimates showed modest differences between treatment states for GPT-5.4 Pro (Δ = +3.7 years; BEFORE: 41.3 ± 10.6; AFTER: 37.6 ± 10.3), suggesting that this model tended to assign lower apparent age to post-treatment images (Table 5). Grok 4.1 showed a smaller difference (Δ = +1.4 years). Gemini 3.1 Pro and Claude Opus 4.6 showed negligible differences between treatment states (Δ = 0.0 and +0.2 years, respectively), indicating that age estimation in these models was not systematically influenced by treatment-associated visual changes.

## 3. Discussion

This study evaluated whether the clinically well-characterized effects of BoNT on facial muscle activity and wrinkle patterns can be detected by contemporary MLLMs based on single-image inference. The results demonstrate marked heterogeneity in model performance across tasks and models, with treatment state classification emerging as the most tractable task for leading models, while region-specific wrinkle detection proved substantially more challenging. The multi-run evaluation design revealed pronounced dissociations between classification accuracy and inter-run reliability that would have been entirely obscured by conventional single-run assessment.

### 3.1. Treatment State Classification

Two of four models achieved perfect accuracy and perfect inter-run agreement for distinguishing pre- from post-treatment images. This performance suggests that these models capture visual features consistent with BoNT-treated faces, potentially including reduced wrinkle depth, altered skin surface texture, and characteristic changes in periorbital and frontalis muscle tone that are well-described clinical correlates of successful BoNT treatment. The remaining two models performed near or below chance level on this task, with one model showing a systematic bias toward classifying images as pre-treatment, as evidenced by near-zero sensitivity for detecting post-treatment images despite high specificity for pre-treatment classification.

The perfect classification achieved by two models warrants cautious interpretation. It cannot be excluded that these models encountered similar image distributions during pre-training, and the balanced design of the dataset (23 BEFORE, 23 AFTER) may have facilitated binary discrimination. The high performance in treatment state classification may partly reflect reliance on global facial cues (e.g., overall skin texture or image-level characteristics) rather than specific detection of localized neuromuscular effects. Nevertheless, the clinical implication is notable: if confirmed in larger, independent datasets, the ability to distinguish treated from untreated faces could support objective treatment outcome documentation in clinical practice [9].

### 3.2. Region-Specific Wrinkle Detection

Detection of wrinkle presence in the forehead, glabella, and periorbital regions proved substantially more difficult than treatment state classification. No model consistently exceeded the majority-class proportion across all three regions, and substantial class imbalance (particularly for periorbital wrinkles, with 78.3% of images classified as wrinkle-absent) complicated interpretation of raw accuracy values. Sensitivity–specificity analysis revealed that two models exhibited systematic overdetection bias, classifying nearly all images as wrinkle-present regardless of ground truth, while the remaining models showed more balanced profiles.

These findings are consistent with the clinical observation that wrinkle assessment in BoNT-treated regions requires nuanced visual interpretation. Forehead wrinkles are produced by frontalis contraction, glabellar lines by corrugator and procerus activation, and periorbital lines by orbicularis oculi contraction [1,2,3,4,5,6,7,8,9]. The absence of dynamic assessment (i.e., models evaluated static images only) likely contributed to the difficulty, as static wrinkle presence depends on skin quality, photo-aging, and residual etching rather than active muscle contraction alone. Future studies incorporating dynamic facial images or video sequences may better capture the muscle-dependent wrinkle patterns that distinguish BoNT-treated from untreated individuals.

### 3.3. Accuracy–Consistency Dissociation

A central finding is the dissociation between classification accuracy and inter-run reliability, a pattern consistently observed in MLLM evaluation across medical domains [19,20,21]. One model achieved the highest overall reliability across all tasks, with substantial to almost perfect Fleiss’ κ values, indicating reproducible case-level responses across repeated evaluations. By contrast, another model that achieved high accuracy on treatment state classification showed only moderate agreement on wrinkle detection tasks, suggesting that correct classifications were not reliably reproduced across runs. One model exhibited even negative κ values, reflecting highly inconsistent responses across runs that effectively preclude clinical utility regardless of aggregate accuracy. In tasks with strong class imbalance, particularly periorbital wrinkle detection, κ values should be interpreted cautiously as agreement metrics may be influenced by prevalence effects [23].

These findings support the broader importance of multi-run evaluation protocols for robust characterization of LLM behavior [19,20,21]. By extension, such approaches should also be considered essential in aesthetic medicine applications, where consistent and reproducible outputs are critical for clinical reliability. Single-run assessments would have identified the best-performing model without revealing its variable consistency on subtasks and would have obscured the systematic unreliability of lower-performing models. For potential clinical applications in BoNT treatment assessment, reliability is arguably as important as accuracy: a system that occasionally produces correct classifications but cannot reproduce them across sessions provides no actionable information for treatment planning.

### 3.4. Exploratory Findings

The exploratory wrinkle severity scores showed that three of four models assigned higher severity ratings to pre-treatment images than to post-treatment images across all three regions, consistent with the expected pattern of reduced wrinkle severity following BoNT treatment. These model-generated scores lack external validation and should be interpreted as reflecting model behavior rather than objective clinical grading. Nevertheless, the directional consistency observed in leading models suggests that MLLMs may have captured visual correlates of wrinkle severity that parallel clinically meaningful distinctions.

Apparent age estimates showed directionally consistent patterns across models, with all four models estimating equal or higher apparent age for pre-treatment images (Δ ranging from 0.0 to +3.7 years). Although the direction is consistent with clinical experience suggesting that successful BoNT treatment is associated with a perceived reduction in apparent age [1,2,3,4,5,6,7,8,9], the small magnitude of the observed differences and the absence of validated age ground truth preclude substantive interpretation.

### 3.5. Comparison with Prior Literature

The present findings extend the emerging literature on MLLM performance in facial assessment. Saeed et al. recently demonstrated that ChatGPT-5 achieved 95.5% agreement with board-certified plastic surgeons for facelift candidacy assessment from standardized photographs [16], establishing that contemporary MLLMs possess clinically relevant facial analysis capabilities. The present study complements this work by evaluating a different and more granular question: whether MLLMs can detect the specific visual effects of BoNT treatment rather than general aging severity.

Prior evaluations of MLLMs in medical imaging have reported heterogeneous results, ranging from near-expert accuracy for visually salient pathology (such as fracture detection) to chance-level performance for tasks requiring nuanced spatial reasoning [10,11,12,24]. Although these evaluations originate from different clinical domains, the underlying technical challenge is comparable: discriminating subtle visual differences in complex anatomical structures from static images. The task-dependent performance hierarchy observed in the present study is consistent with this pattern: treatment state classification, which likely relies on global facial cues associated with BoNT-induced muscle relaxation, was performed well by leading models, whereas region-specific wrinkle detection, which requires localized visual discrimination of individual muscle group effects, proved more challenging.

### 3.6. Clinical Implications

From a clinical perspective, the present findings indicate that current MLLMs are not suitable for meaningful use in BoNT treatment assessment. Although leading models were able to distinguish pre- from post-treatment images, performance in region-specific wrinkle detection was inconsistent, and inter-run variability further limits reproducibility. These limitations preclude clinically relevant application at this stage.

It should be emphasized that the use of AI in aesthetic treatment assessment remains in its infancy. In routine practice, an experienced clinician trained in BoNT application not only identifies treatment-related facial changes with high accuracy but does so more reliably and substantially faster than current AI systems. A direct quantitative comparison of evaluation time is inherently limited by the fundamentally different workflows of clinical assessment and AI-based inference. Within the workflow of this study, however, repeated model queries across independent sessions required considerably more time per case without providing additional clinically actionable information. Under these conditions, the cost–benefit ratio of AI-assisted BoNT outcome evaluation appears unfavorable.

Importantly, the present study used expert clinical assessment as its reference standard, and the results consistently demonstrate that human evaluation remains the benchmark against which AI performance must be measured.

Accordingly, MLLMs should currently be regarded as research-stage tools rather than clinically applicable systems. While they may support standardized outcome documentation in controlled research settings, their present performance does not meet the requirements for independent clinical use. Nevertheless, given the rapid evolution of multimodal AI systems, future improvements in visual reasoning and consistency may enable more reliable assessment of BoNT-induced facial changes. Until such validation is achieved, any clinical integration should remain strictly supervised.

### 3.7. Limitations

Several limitations should be considered when interpreting these results.

1. The dataset comprised 46 images, which, while sufficient for initial evaluation, limits the precision of performance estimates. Descriptively meaningful accuracy differences between models should be interpreted with caution.
2. The evaluation was limited to static facial photographs. BoNT effects are most prominent during active facial expression, and the absence of dynamic assessment likely reduced the discriminative signal available to the models.
3. Detailed treatment parameters (e.g., BoNT formulation, dosing per region) were not available from the source dataset and therefore could not be analyzed, which may have influenced the observed variability in model performance.
4. All models were accessed via their official web interfaces using default inference settings. Sampling parameters, temperature, and other decoding configurations were not controlled and may have contributed to inter-run variability.
5. It cannot be excluded that some images were included in the training datasets of one or more evaluated models. However, the substantial inter-run variability observed across most models argues against pure memorization.
6. The use of a single curated dataset introduces the possibility that models relied on dataset-specific visual characteristics (e.g., lighting conditions, image composition, or post-processing) rather than exclusively on biologically meaningful features associated with BoNT effects.
7. The evaluation employed a zero-shot prompting strategy without task-specific instructions, chain-of-thought reasoning, or few-shot examples. Alternative prompting strategies may yield different performance profiles.
8. Wrinkle severity scores and age estimates were treated as exploratory endpoints because no external reference standard was available. These variables reflect model behavior rather than objective clinical measurements.
9. The dataset included images from a single publicly available source. Generalizability to diverse clinical populations, imaging conditions, and treatment protocols requires validation in independent cohorts.
10. MLLM capabilities evolve rapidly through provider-side updates. The present results reflect model versions available in March 2026 and represent a cross-sectional snapshot that may not generalize to future iterations.

## 4. Conclusions

The characteristic visual effects of BoNT on upper-face wrinkle patterns can be partially captured by contemporary multimodal AI systems under controlled conditions. While treatment state classification was feasible for leading models, region-specific assessment of wrinkle patterns, representing a more direct surrogate of underlying muscle activity, remained inconsistent and model-dependent. The observed dissociation between accuracy and inter-run reliability underscores that single-run evaluations substantially overestimate model robustness.

These findings position current MLLMs as exploratory tools for the quantitative assessment of BoNT-induced facial changes rather than clinically reliable systems. At present, their performance does not support independent clinical application. However, the conceptual approach of using AI-based analysis of wrinkle patterns as a non-invasive surrogate of neuromuscular modulation may provide a foundation for future, more robust methods of objective BoNT outcome assessments.

## 5. Materials and Methods

### 5.1. Study Design and Objectives

This observational study evaluated the diagnostic performance of four MLLMs in detecting visual patterns consistent with BoNT–induced facial changes using a repeated-run inference framework. The study was designed to characterize classification accuracy, inter-run reliability (i.e., agreement across repeated inference runs of the same model), and response stability across multiple independent inference runs rather than to establish clinical validation or population-level diagnostic performance.

The study had three primary objectives: (1) to quantify classification accuracy across five independent inference runs per image for four distinct tasks; (2) to assess inter-run reliability across repeated runs using Fleiss’ kappa (κ) [23]; and (3) to evaluate the relationship between classification accuracy and response consistency across models and tasks.

### 5.2. Dataset and Image Selection

A total of 46 facial images (23 before treatment, 23 after botulinum neurotoxin treatment) were selected from a publicly available, curated facial image dataset [25], licensed under a Creative Commons Attribution–NonCommercial–NoDerivatives 4.0 International (CC BY-NC-ND 4.0) license. The dataset comprised standardized facial photographs of adult individuals who had undergone BoNT treatment of the upper face, including the forehead, glabella, and periorbital regions. Specific treatment details (e.g., BoNT formulation, dosing per region, and injection technique) were not available from the dataset source and could not be controlled or standardized within the study design. This reflects real-world clinical conditions, where patients frequently present after prior treatments performed elsewhere, often without reliable documentation of toxin type, dosage, or injection pattern, thereby limiting standardization of treatment-related effects. Images were selected to represent a balanced distribution of pre- and post-treatment states. Due to licensing restrictions, the original images cannot be redistributed by the authors.

Ground truth labels were established at two levels. Treatment state (BEFORE vs. AFTER) was derived from the dataset structure. Region-specific wrinkle presence (binary: 0 = absent, 1 = present) for the forehead, glabella, and periorbital regions was annotated by consensus among multiple board-certified plastic surgeons with extensive clinical experience in botulinum neurotoxin treatment. The class distribution showed moderate to substantial imbalance favoring the absent class: forehead (65.2% absent), glabella (73.9% absent), and periorbital (78.3% absent). Treatment state was balanced (50.0%). The standardized evaluation prompt is provided in the Supplementary Materials.

### 5.3. Models Under Evaluation

Four contemporary MLLMs were evaluated to represent a diverse spectrum of proprietary AI platforms:

- GPT-5.4 Pro (OpenAI, San Francisco, CA, USA; proprietary)
- Grok 4.1 (xAI, San Francisco, CA, USA; proprietary)
- Gemini 3.1 Pro (Google DeepMind, Mountain View, CA, USA; proprietary)
- Claude Opus 4.6 (Anthropic, San Francisco, CA, USA; proprietary)

All models were accessed via their official web interfaces using default inference settings. No application programming interface (API)-level parameter modifications (e.g., temperature, top-p, or decoding strategies) were applied. This approach was chosen to approximate real-world usage conditions and to reflect how these systems are typically accessed by clinicians and researchers in practical settings. Model versions reflect the publicly available system state at the time of data collection in March 2026.

### 5.4. Prompting Strategy and Inference Protocol

Each model received five inference runs per image using an identical, standardized zero-shot prompt (see Supplementary Material). Critically, each image was evaluated independently: the model was not shown paired before/after images, did not know that images belonged to the same individual, and received no information about treatment history. The task was single-image inference only.

The prompt instructed the model to analyze the provided facial image and report: (1) treatment state classification (BEFORE or AFTER); (2) binary wrinkle presence for three regions (forehead, glabella, periorbital; 0 or 1); (3) wrinkle severity rating for each region (ordinal scale, 0–4); and (4) estimated apparent age (integer). To ensure independence between runs, each inference was conducted in a fresh session without prior conversational context. No conversational memory, feedback, or adaptive prompting was permitted.

### 5.5. Outcome Definitions

Primary outcomes included: (1) classification accuracy for each of the four binary tasks (treatment state, forehead wrinkle detection, glabella wrinkle detection, and periorbital wrinkle detection), defined as the mean accuracy across five independent inference runs; (2) sensitivity and specificity for each binary task, with the positive class defined as AFTER for treatment state and as wrinkle present (1) for regional detection tasks; and (3) inter-run reliability for each binary task, quantified using Fleiss’ κ to assess agreement across repeated runs [23].

Secondary outcomes included: (1) wrinkle severity scores (ordinal, 0–4), reported descriptively as model-generated predictions stratified by true treatment state; and (2) apparent age estimates, reported descriptively without ground truth validation. Wrinkle severity scores and age estimates were treated as exploratory endpoints because no external reference standard was available for these variables.

### 5.6. Conceptual Framework

Wrinkle presence and severity were interpreted as visual surrogate markers reflecting underlying facial muscle activity affected by BoNT. This study did not measure neuromuscular blockade directly. Rather, it evaluated whether MLLMs can recognize visual patterns consistent with BoNT-induced changes in facial muscle activity based on single-image inference. In this context, visual features are interpreted as indirect indicators of underlying facial muscle activity, consistent with established links between neuromuscular function and facial appearance [26,27].

### 5.7. Statistical Analysis

All statistical analyses were performed using Python 3.12 (Python Software Foundation, Wilmington, DE, USA) with standard statistical and data visualization libraries. Classification accuracy for each binary task was summarized descriptively as the mean accuracy across five inference runs, with the corresponding range (minimum–maximum) and standard deviation reported to illustrate inter-run variability. Sensitivity and specificity were computed per model per task per run and summarized as Mean ± SD across runs.

Inter-run reliability was assessed using Fleiss’ κ [23], computed on raw model outputs (BEFORE/AFTER or 0/1) rather than on correct/incorrect classifications, reflecting consistency of the model’s actual responses rather than its error patterns. κ values were interpreted according to the conventional Landis–Koch thresholds [22]: <0.20 slight, 0.21–0.40 fair, 0.41–0.60 moderate, 0.61–0.80 substantial, and 0.81–1.00 almost perfect agreement. Fleiss’ κ is sensitive to prevalence effects and was interpreted with this limitation in mind [28,29].

For each binary task, a majority-class proportion was calculated to provide a reference threshold for evaluating model performance. A naive classifier always predicting the most frequent class would achieve the majority-class proportion as its accuracy, representing the minimum meaningful performance benchmark.

Exploratory wrinkle severity scores were reported as Mean ± SD, stratified by true treatment state (BEFORE vs. AFTER), with the difference (Δ = Mean (BEFORE) − Mean (AFTER)) indicating the direction of score change. Age estimates were analyzed descriptively using the same stratification. No inferential statistical testing was performed on exploratory variables, as these lacked external ground truth validation. Inter-run reliability for ordinal severity scores was computed using Fleiss’ κ with the caveat that the ordered nature of categories was not accounted for [8,23].

## Figures and Tables

**Table 1:** Dataset Composition and Class Distribution.

Task	Absent (n)	Present (n)	Total	Majority-Class Proportion (%)
Treatment state classification	23 (BEFORE)	23 (AFTER)	46	50.0
Wrinkle detection: forehead	30	16	46	65.2
Wrinkle detection: glabella	34	12	46	73.9
Wrinkle detection: periorbital	36	10	46	78.3

N = 46 facial images (23 before, 23 after BoNT treatment), evaluated by 4 multimodal large language models across 5 independent runs each (920 assessments per task). The majority-class proportion reflects the expected accuracy of a naive classifier always predicting the most frequent class.

**Table 2:** Classification Performance Across Primary Tasks.

Task	Model	Accuracy, % (Range)	Sensitivity (%)	Specificity (%)	κ
**Treatment state (before/after)—majority-class proportion: 50.0%**					
	GPT-5.4 Pro	63.0 ± 6.9 (52.2–69.6)	38.3 ± 9.9	87.8 ± 9.4	0.389
	Grok 4.1	48.3 ± 4.2 (41.3–52.2)	0.9 ± 1.9	95.7 ± 7.5	−0.027
	Gemini 3.1 Pro	100.0 ± 0.0 (100.0–100.0)	100.0 ± 0.0	100.0 ± 0.0	1.000
	Claude Opus 4.6	100.0 ± 0.0 (100.0–100.0)	100.0 ± 0.0	100.0 ± 0.0	1.000
**Forehead wrinkle detection—majority-class proportion: 65.2%**					
	GPT-5.4 Pro	57.0 ± 5.2 (50.0–63.0)	80.0 ± 10.3	44.7 ± 6.5	0.548
	Grok 4.1	35.7 ± 1.2 (34.8–37.0)	97.5 ± 5.6	2.7 ± 2.8	−0.027
	Gemini 3.1 Pro	71.3 ± 18.3 (39.1–82.6)	96.2 ± 3.4	58.0 ± 29.4	0.553
	Claude Opus 4.6	64.8 ± 3.6 (58.7–67.4)	73.8 ± 2.8	60.0 ± 5.8	0.808
**Glabella wrinkle detection—majority-class proportion: 73.9%**					
	GPT-5.4 Pro	71.3 ± 6.8 (63.0–78.3)	98.3 ± 3.7	61.8 ± 8.6	0.606
	Grok 4.1	27.0 ± 2.5 (23.9–30.4)	96.7 ± 4.6	2.4 ± 3.8	−0.027
	Gemini 3.1 Pro	82.6 ± 7.5 (69.6–87.0)	76.7 ± 7.0	84.7 ± 12.0	0.636
	Claude Opus 4.6	77.8 ± 2.4 (76.1–80.4)	58.3 ± 11.8	84.7 ± 6.7	0.732
**Periorbital wrinkle detection—majority-class proportion: 78.3%**					
	GPT-5.4 Pro	50.0 ± 6.5 (43.5–60.9)	90.0 ± 12.2	38.9 ± 6.5	0.604
	Grok 4.1	25.2 ± 3.6 (21.7–30.4)	90.0 ± 10.0	7.2 ± 6.7	−0.025
	Gemini 3.1 Pro	60.0 ± 10.5 (43.5–71.7)	82.0 ± 20.5	53.9 ± 16.6	0.501
	Claude Opus 4.6	82.6 ± 4.3 (76.1–87.0)	64.0 ± 18.2	87.8 ± 9.9	0.601

Values: Mean ± SD (%) across 5 independent runs. Sensitivity and specificity are reported with respect to the positive class: AFTER for treatment state; wrinkle present (1) for regional detection. Fleiss’ κ computed on raw model outputs (BEFORE/AFTER or 0/1), not correct/incorrect. κ < 0 indicates agreement below chance level; 0.01–0.20 slight; 0.21–0.40 fair; 0.41–0.60 moderate; 0.61–0.80 substantial; and 0.81–1.00 almost perfect (Landis & Koch, 1977) [22,23].

**Table 3:** Summary Performance Across Binary Classification Tasks.

Model	Mean Accuracy Across Tasks (%)
GPT-5.4 Pro	60.3
Grok 4.1	34.0
Gemini 3.1 Pro	78.5
Claude Opus 4.6	81.3

Mean accuracy across the four predefined binary classification tasks (treatment state classification, forehead wrinkle detection, glabella wrinkle detection, and periorbital wrinkle detection), each task equally weighted, not pooled.

**Table 4:** Exploratory Wrinkle Severity Scores by Treatment State.

Region	Model	BEFORE (Mean ± SD)	AFTER (Mean ± SD)	Δ	κ
**Forehead**					
	GPT-5.4 Pro	1.99 ± 0.97	0.67 ± 0.88	+1.32	0.388
	Grok 4.1	2.43 ± 0.71	2.36 ± 0.61	+0.07	0.007
	Gemini 3.1 Pro	1.97 ± 0.77	0.23 ± 0.42	+1.75	0.449
	Claude Opus 4.6	1.59 ± 0.90	0.20 ± 0.40	+1.39	0.781
**Glabella**					
	GPT-5.4 Pro	1.84 ± 1.10	0.52 ± 0.74	+1.32	0.383
	Grok 4.1	2.43 ± 0.71	2.38 ± 0.57	+0.05	−0.040
	Gemini 3.1 Pro	1.10 ± 1.05	0.01 ± 0.09	+1.10	0.484
	Claude Opus 4.6	0.96 ± 1.08	0.03 ± 0.18	+0.92	0.719
**Periorbital**					
	GPT-5.4 Pro	1.66 ± 0.86	0.67 ± 0.78	+0.99	0.402
	Grok 4.1	2.06 ± 0.78	1.79 ± 0.76	+0.27	−0.051
	Gemini 3.1 Pro	1.35 ± 1.02	0.28 ± 0.52	+1.07	0.414
	Claude Opus 4.6	0.34 ± 0.63	0.23 ± 0.42	+0.11	0.521

No ground truth available. Scores (ordinal, 0–4) are model-generated predictions stratified by true treatment state. Δ = Mean (BEFORE) − Mean (AFTER); positive values indicate higher predicted severity in pre-treatment images. Fleiss’ κ computed on ordinal categories; the ordered nature is not accounted for.

**Table 5:** Exploratory Apparent Age Estimates by Treatment State.

Model	BEFORE (Mean ± SD)	AFTER (Mean ± SD)	Δ (Years)
GPT-5.4 Pro	41.3 ± 10.6	37.6 ± 10.3	+3.7
Grok 4.1	42.3 ± 8.7	40.9 ± 8.4	+1.4
Gemini 3.1 Pro	40.9 ± 9.9	40.9 ± 10.0	0.0
Claude Opus 4.6	38.5 ± 11.4	38.4 ± 11.4	+0.2

No ground truth available. Age estimates (integer years) are approximate model outputs without external validation. Δ = Mean (BEFORE) − Mean (AFTER). Differences reflect model behavior, not biological change.

## Data Availability

The data used in this study are publicly available from an online repository (https://www.kaggle.com/datasets/trainingdatapro/botox-injections-before-and-after; accessed on 4 February 2026) under a Creative Commons Attribution-NonCommercial-NoDerivatives 4.0 International license. Due to licensing restrictions, the images cannot be redistributed by the authors. The standardized evaluation prompt is provided in the Supplementary Materials.

## Supplementary Materials

The following supporting information can be downloaded at: https://www.mdpi.com/article/10.3390/toxins18040188/s1, Supplementary File S1: Standardized evaluation prompt used for model assessment.

## Abbreviations

The following abbreviations are used in this manuscript:

AI	Artificial Intelligence
API	Application Programming Interface
BoNT	Botulinum Neurotoxin
DL	Deep Learning
GenAI	Generative Artificial Intelligence
IME	Index of Muscular Equilibrium
LLM	Large Language Model
MLLM	Multimodal Large Language Model
SD	Standard Deviation
κ (Kappa)	Fleiss’ Kappa
