# Supplementary material for: AI-Based Quantification of Botulinum Neurotoxin-Induced Facial Changes: Wrinkle Reduction, Region-Specific Effects, and Functional Correlates of Facial Muscle Activity
Source: Toxins (Basel). 2026 Apr 15;18(4):188. doi: 10.3390/toxins18040188 (PMC13119919; doi:10.3390/toxins18040188)
Supplement: Supplementary file 1 [file toxins-18-00188-s001.zip › toxins-4237730-supplementary.pdf]

Supplementary Materials: AI-Based Quantification of Botulinum Neurotoxin–Induced Facial Changes: Wrinkle Reduction, Region-Specific Effects, and Functional Correlates of Facial Muscle Activity

Ibrahim Güler, Armin Kraus, Gerrit Grieb and Henrik Stelling

You are evaluating a single facial image for signs consistent with botulinum toxin treatment.

Tasks:

1. Classify the image as more likely:
BEFORE = consistent with no treatment
AFTER = consistent with post botulinum toxin treatment

2. Assess wrinkle presence (binary):
0 = no clearly visible static wrinkles
1 = clearly visible static wrinkles

Regions:
- forehead (frontalis region)
- glabella (corrugator/procerus region)
- periorbital (lateral canthal region)

3. Rate wrinkle severity (0–4):
0 = none
1 = mild
2 = moderate
3 = severe
4 = very severe

4. Estimate apparent age:
INTEGER (range 18–90)

STRICT OUTPUT RULES (MANDATORY):

- Output EXACTLY 8 LINES
- NO additional text
- NO explanations

- Use EXACT labels
- Use ":" as separator
- Use ONLY the allowed values

OUTPUT FORMAT (FIXED):

state: BEFORE or AFTER
forehead_binary: 0 or 1
glabella_binary: 0 or 1
periorbital_binary: 0 or 1
forehead_score: 0 or 1 or 2 or 3 or 4
glabella_score: 0 or 1 or 2 or 3 or 4
periorbital_score: 0 or 1 or 2 or 3 or 4
age: INTEGER
